# Altered tear fluid protein expression in persons with mild cognitive impairment

**DOI:** 10.1177/13872877251413291

**Published:** 2026-01-20

**Authors:** Virve Kärkkäinen, Toni Saari, Minna Rusanen, Hannu Uusitalo, Ville Leinonen, Bernd Thiede, Anne M. Koivisto, Kai Kaarniranta, Tor P. Utheim

**Affiliations:** 1 NeuroCenter, Neurology, Kuopio University Hospital, Kuopio, Finland; 2 NeuroCenter, Neurosurgery, Kuopio University Hospital, Kuopio, Finland; 3 Neurosurgery, Institute of Clinical Medicine, School of Medicine, University of Eastern Finland, Kuopio, Finland; 4 Institute for Molecular Medicine Finland (FIMM), HiLIFE, University of Helsinki, Helsinki, Finland; 5 Geriatric Center, Wellbeing Services Country of North Karelia, Joensuu, Finland; 6 Neurology, Institute of Clinical Medicine, School of Medicine, University of Eastern Finland, Kuopio, Finland; 7 Eye and Vision Research, Faculty of Medicine and Health Technology, Tampere University, Tampere, Finland; 8 Department of Biosciences, University of Oslo, Oslo, Norway; 9 Department of Geriatrics, Helsinki University Hospital and Department of Neurosciences, University of Helsinki, Helsinki, Finland; 10 Department of Ophthalmology, Institute of Clinical Medicine, School of Medicine, University of Eastern Finland and Kuopio University Hospital, Kuopio, Finland; 11 Department of Ophthalmology, Oslo University Hospital, Oslo, Norway; 12 Department of Medical Biochemistry, Oslo University Hospital, Oslo, Norway; 13 Department of Ophthalmology, Sørlandet Hospital Trust, Arendal, Norway

**Keywords:** Alzheimer's disease, biomarkers, inflammation, mild cognitive impairment, oxidative stress, protein function, proteomics, tear fluid

## Abstract

**Background:**

Tear fluid (TF) is a protein-rich fluid reported to reflect pathophysiological changes in several neurodegenerative diseases, including Alzheimer's disease. TF proteins are increasingly being considered as putative biomarker candidates to help in the diagnosis of disease. However, little information is available on TF protein changes in persons with mild cognitive impairment (MCI).

**Objective:**

This study aimed to determine alterations in the expression of proteins in TF collected from persons with MCI compared with cognitively healthy controls.

**Methods:**

We analyzed data from 54 study participants, including 34 controls (mean age, 71 years; mean Mini-Mental State Examination [MMSE] score ± standard deviation, 28.9 ± 1.4) and 20 persons with MCI (mean age, 71 years; mean MMSE score, 27.1 ± 1.9). All participants underwent cognitive, neurological, and ophthalmological examinations. TF was collected using Schirmer strips and evaluated using mass spectrometry-based proteomics and label-free quantification.

**Results:**

The expression of 33 TF proteins involved in oxidative stress, clearance mechanism, cytoskeleton stability, and inflammation were altered in persons with MCI compared with controls (*p* ≤ 0.05).

**Conclusions:**

Our findings reveal that numerous cellular stress-related biomarker candidate proteins are upregulated or downregulated in the TF of persons with MCI, a condition that may increase the risk of developing AD or other memory disorder. These data encourage TF protein studies in neurodegenerative diseases and TF provides an additive source of biomarkers for early diagnostics of memory diseases.

## Introduction

Typical of many progressive neurodegenerative diseases, such as Alzheimer's disease (AD), is progression in the brain for many years, or even decades, before the first clinical symptoms appear. The period before the onset of clinical symptoms is referred to as the preclinical stage.^1^ The next stage in disease progression is mild cognitive impairment (MCI), which is an intermediate state between normal aging and the continuum of declining memory. In the MCI stage, the individual begins to experience early signs of cognitive decline and has an increased risk of progression to dementia.^1,2^ After a person is diagnosed with MCI, in some of them MCI progresses to AD dementia, and death typically occurs within 3 to 9 years.^3^

An effective cure for AD is not yet available. Current medical treatments can only alleviate symptoms and slightly delay disease progression, particularly when initiated in the early stages.^2,4^ Diagnosis of neurodegenerative diseases is challenging when the patient's symptoms are mild and disease-specific symptoms absent. This may lead to disease progression, late diagnosis, and a weak response to available medication.^5^

New biomarker candidates that could be used to distinguish neurodegenerative diseases from each other in their early stages are being studied intensively.^6^ Tear fluid (TF) has become an optimal source for researching new biomarker molecules because it is easy to collect and protein-rich, containing more than 1500 proteins.^7–11^ Many TF proteins are either produced by the lacrimal gland or reach the eyes from the serum via the conjunctival capillaries, making them a potential source of biomarker candidates for systemic disorders, such as AD.^10^ Furthermore, the composition of TF proteins seems to reflect several factors, including aging and various diseases.^7,8,12^

An optimal biomarker candidate for neurodegenerative diseases should reveal the disease at the beginning of its progression, in early MCI stage or even in risk stage. To date, only a few TF protein studies have been conducted in persons with MCI. These studies showed that AD-specific proteins are secreted into the TF and that the functions of several other TF proteins are altered in the MCI stage.^8,13,14^ However, further studies are needed to clarify how the functions of TF proteins change during the MCI stage and whether these changes are indicative of specific diseases.

The aim of this study was to compare the protein expression levels in TF between persons with MCI and cognitively healthy controls using liquid chromatography/mass spectrometry (LC/MS).

## Methods

### Study design and protocol

For this cross-sectional study, we recruited a total of 54 participants aged >60 years from the Brain Research Unit of the University of Eastern Finland and from the memory clinic of Kuopio University Hospital NeuroCenter. The study participants were divided into the two study groups based on the results of their memory tests as described below: 34 were considered cognitively healthy and 20 were classified as having MCI but no diagnosed memory disease. The exclusion criteria included the individuals who had already been diagnosed with a memory disorder, diabetes, or an eye disease, such as ocular surface disease, glaucoma, or age-related macular degeneration.

At the study appointments, all study participants underwent the same interviews and tests. Comprehensive demographic information was obtained from participants and family members, including medical history, medication, and family history of AD (Table 1). The Clinical Dementia Rating (CDR) scale was administered to all participants.^15^ Neurological examinations were carried out and participants with any signs of upper motor neuron compromise, parkinsonism, balance abnormalities, or ataxia were excluded from the study. The Finnish version of the CERAD neuropsychological test battery, including the Mini-Mental State Examination (MMSE), was administered to all participants.^16^

**Table 1.** Demographics of the control (CO) and mild cognitive impairment (MCI) study groups.

Characteristic	CO	MCI	*p*
Number of participants	34	20	
Age, y	71 (5.3)	71 (6.6)	0.897
Female	19 (56%)	9 (45%)	0.440
MMSE score	28.6 (1.4)	27.1 (1.9)	**0**.**002**
MMSE range	25–30	23–30	N/A
Family history of memory diseases	19 (57.6%)	9 (45.0%)	0.496
Education, y	12.55 (4.3)	10.65 (3.7)	0.054
BMI, kg/m^2^	25.3 (2.6)	25.5 (3.5)	0.786
Coronary artery disease	3 (8.8%)	3 (15%)	0.486
Hypercholesterolemia medication	14 (41.2%)	6 (30.0%)	0.411
Hypertension medication	19 (55.9%)	11 (55.0%)	0.950
*APOE* allele 4 carriers	44.1 (15%)	38.9 (7%)	0.717
Memory problems	1 (2.9%)	11 (55%)	**0**.**001**

Values are presented as n (%) or mean (SD) unless otherwise indicated. Bold indicates significant differences between groups. SD: standard deviation; MMSE: Mini-Mental State Examination (range 0–30); *APOE*: apolipoprotein E; N/A: not applicable.

If CERAD results were in the normal range (mean MMSE score ± standard deviation [SD], 28.6 ± 1.4) and the participant had no decline in daily activities based on demographic and CDR interviews (i.e., CDR 0), they were assigned to the control group. If the CERAD results indicated mild decline (mean MMSE score ± SD, 27.1 ± 1.9) and the participant had very mild decline in daily activities (i.e., CDR 0–0.5), they were assigned to the MCI group.

### APOE genotyping

Genomic DNA was extracted from venous blood samples using the QIAamp DNA Blood Mini Kit (QIAGEN). *APOE* alleles were determined using TaqMan genotyping assays (Applied Biosystems, Foster City, CA, USA) for two single nucleotide polymorphisms (rs429358 and rs7412) and an allelic discrimination method on the ABI 7000 platform.^17^

### Eye examination

Slit-lamp examination was performed using a CSO biomicroscope (Costruzuzione Strumenti Oftalmici, Firenze, Italy). Tear film stability was assessed based on tear film break-up time, and Schirmer test strips were used to measure TF production.^18,19^ Lid margin and bulbar conjunctival redness were scored according to the Institute of Eye Research grading scales (very slight, 0; mild, 1; moderate, 2; or severe, 3).^18^ Corneal and conjunctival fluorescein staining patterns were scored using the Oxford grading scale from 1 (absent) to 5 (marked).^19^

### Tear fluid collection and sample preparation

TF samples were collected using Schirmer strips (Tear Touch; Madhu Instruments) without anesthesia. TF samples were collected from all participants at the same time of day to avoid any influence of collection timing on protein composition. The strips were placed under the lower eyelid for 5 min and then into 1.5-ml Eppendorf tubes for storage at −80°C until further processing.^11,20^

### In-solution protein digestion

Proteins were eluted from the Schirmer strips by 200 µl phosphate-buffered saline and precipitated with cold acetone. In-solution digestion of the proteins was performed with 2 µg trypsin GOLD (Promega, Madison, WI, USA) after reductive alkylation using DTT and iodoacetamide.

### Protein identification and label-free quantification

Tryptic peptides were purified using 10 µl OMIX-C18 micro-SPE pipette tips (Agilent, Santa Clara, CA, USA). The purified peptides were injected into the LC/MS system, which consisted of a timsTOF Pro (Bruker Daltonik, Bremen, Germany) coupled online to a nanoElute nanoflow liquid chromatography system (Bruker Daltonik, Bremen, Germany) via a CaptiveSpray nanoelectrospray ion source. The LC/MS data were searched against the human Uniprot database (20,431 entries) using PEAKS X + software version 10.5 (Bioinformatics Solutions, Waterloo, ON, Canada). A false discovery rate (FDR) of 1% was applied to the data sets.

For label-free quantification using PEAKS, ID-directed label-free quantification was performed with outlier removal. The following parameters were applied regarding peptide features: quality ≥ 4, peptide ID count per group ≥ 1, and detected in ≥ 1 sample per group. The following parameters were applied to the proteins: FDR ≤ 5%, fold change ≥ 2, and significance in analysis of variance with ≥ 2 peptides. The significance score was calculated as the −10log10 of the *p*-value obtained in significance testing.

### Statistical analysis

Statistical analyses were performed using SPSS 22 software (SPSS Inc., Chicago, IL, USA). A *p-*value <0.05 was considered significant. Results regarding demographic data, CERAD test/MMSE, and ocular data (Table 2) are presented as the mean ± SD or as the number and proportion of cases. Comparisons were made using T-test. Group differences in continuous ocular data were expressed as the mean difference with 95% confidence intervals.

**Table 2.** Ocular data for the control (CO) and mild cognitive impairment (MCI) groups.

Measure	CO *n* *=* *34*	MCI *n* *=* *20*	*p*	Mean difference (95% CI)
Schirmer, mm in 5 min, right eye	9.28 (6.9)	8.4 (7.7)	0.648	0.93 (−3.1 to 4.9)
Schirmer, mm in 5 min, left eye	9.03 (6.1)	10.75 (7.4)	0.352	−1.7 (−5.4 to 1.9)
TBUT, right eye	5.54 (3.1)	5.45 (3.4)	0.919	0.15 (−1.7 to 1.9)
TBUT, left eye	5.6 (3.2)	5.45 (3.4)	0.872	0.93 (−1.7 to 2.0)
Conjunctival redness, right eye			0.389	
0 = no redness	0 (0%)	0 (0%)		
1 = mild redness	21 (58.3%)	14 (70.0%)		
2 = moderate redness	15 (41.7%)	6 (30.0%)		
3 = severe redness	0 (0%)	0 (0%)		
Conjunctival redness, left eye			0.631	
0 = no redness	0 (0%)	0 (0%)		
1 = mild redness	21 (58.3%)	13 (65.0%)		
2 = moderate redness	15 (41.7%)	7 (35.0%)		
3 = severe redness	0 (0%)	0 (0%)		
Lid margin redness, right eye			0.364	
0 = no redness	0 (0%)	0 (0%)		
1 = mild redness	25 (69.4%)	10 (50.0%)		
2 = moderate redness	9 (25.0%)	10 (50.0%)		
3 = severe redness	2 (5.6%)	0 (0%)		
Lid margin redness, left eye			0.667	
0 = no redness	1 (2.8%)	0 (0%)		
1 = mild redness	24 (66.7%)	12 (60.0%)		
2 = moderate redness	9 (25.0%)	8 (40.0%)		
3 = severe redness	2 (5.6%)	0 (0%)		
Conjunctival fluorescein staining, right eye			0.440	
0 = absent/very slight	2 (5.6%)	5 (25.0%)		
1 = mild	29 (80.6%)	11 (55.0%)		
2 = moderate	5 (13.9%)	4 (20%)		
Conjunctival fluorescein staining, left eye			0.590	
0 = absent/very slight	2 (5.6%)	3 (15%)		
1 = mild	28 (77.8%)	13 (65%)		
2 = moderate	6 (16.7%)	4 (20%)		
Cornea fluorescein staining, right eye			0.772	
0 = absent/very slight	16 (44.4%)	9 (45.0%)		
1 = mild	14 (38.9%)	9 (45.0%)		
2 = moderate	6 (16.7%)	2 (10.0%)		
Cornea fluorescein staining, left eye			0.247	
0 = absent/very slight	17 (47.2%)	9 (45%)		
1 = mild	12 (33.3%)	10 (50%)		
2 = moderate	7 (19.4%)	1 (5%)		

Results are presented as measurement results (SD) or number (%). CI: confidence interval; SD: standard deviation; TBUT: tear film break-up time.

## Results

### Demographic data

The demographics of the participants are presented in Table 1. The two study groups differed significantly in their mean MMSE scores (*p* < 0.001). In the interview, study participants were asked whether they felt they had any kind of memory problems in their life. In the control group, 2.9% reported that they may have some kind of memory decline, whereas 55% in the MCI group reported potential memory decline.

### Ocular data

TF production (Schirmer test) was measured in both eyes. The study groups did not significantly differ in TF production, tear film break-up time, lid margin redness, conjunctival redness, or ocular surface staining (Table 2).

### Proteomics data

We identified a total of 33 proteins in the TF samples that were significantly upregulated or downregulated (fold change >2) in the MCI group compared to the control group. These proteins could be categorized into groups based on their function (Table 3). The decrease or increase in the amount of protein in MCI samples was relative to the control samples, with no difference being a ratio of 1, significant decrease ratio≤0.5, and significant increase ratio≥2.0.

**Table 3.** Altered expression of tear fluid proteins in participants with mild cognitive impairment (MCI) compared with cognitively healthy controls (CO).

Name of protein by function	Gene name	CO:MCI ratio	Significance score*
OXIDATIVE STRESS Upregulated			
Nucleosome assembly protein 1-like	NP1L4	8.23	38
Ribonuclease T2	RNASET2	2.96	24
Ribonuclease 4	RNASE4	2.72	23
Gamma-butyrobetaine dioxygenase	BBOX1	2.66	59
Cystatin-C	CST3	2.55	98
Glycogen phosphorylase liver form	PYGL	2.35	95
Triosephosphate isomerase	TPIS	2.17	77
Downregulated			
Cystatin-SN	CST1	0.12	74
Mitochondrial peroxiredoxin-5	PRDX5	0.15	121
CYTOSKELETON Upregulated			
Myosin light polypeptide 6	MYL6	2.81	45
Destrin	DSTN	2.38	74
Downregulated			
Tropomyosin alpha-4 chain	TPM4	0.38	90
Protein control quality system Upregulated			
Proteasome activator complex subunit 2	PSME2	2.01	54
Ubiquitin thioesterase	OTUB1	2.0	41
Downregulated			
Heat shock protein 90 kDa	HSP90AA1	0.17	31
Platelet-activating factor acetylhydrolase IB subunit alpha	PAFAH1B1	0.3	105
Rho GDP-dissociation inhibitor	ARHGDIB	0.49	32
Heat shock protein 70 kDa	HSPA4	0.41	39
NSFL1 cofactor p47	NSF1C	0.45	43
INFLAMMATION Upregulated			
SPARC-like protein 1	SPARCL1	2.79	37
Immunoglobulin J chain	JCHAIN	2.42	57
Chitinase-3-like protein	CHI3L2	2.38	63
Mucin-5AC	MUC5AC	2.38	39
Immunoglobulin heavy variable 5–51	IGHV5-51	2.18	39
Glucose-6-phosphate isomerase	GPI	2.16	48
Alpha-1-antitrypsin	SERPINA1	2.12	43
Fibrinogen alpha chain	FGA	2.11	89
Downregulated			
Prolactin-inducible protein	PIP	0.03	153
Mammaglobin B	SCGB2A1	0.05	117
Immunoglobulin kappa light chain	IGK	0.27	58
Lipocalin-1	LCN1	0.29	24
Lysozyme C	LYZ	0.35	42
Deleted in malignant brain tumors 1 protein	DMBT1	0.32	33

* The significance score was calculated as the −10log10 of the *p*-value. The differences were considered significant (*p* ≤ 0.05, T-test) if the ratio was <0.5 or >2.0.

Expression levels of TF proteins are involved in oxidative stress, protein synthesis, and energy metabolism that were different between the study groups included nucleosome assembly protein 1-like (NP1L4), ribonuclease T2 (RNASET2), ribonuclease 4 (RNASE4), gamma-butyrobetaine dioxygenase (BBOX1), cystatin-C (CST3), cystatin-SN (CST1), triosephosphate isomerase (TPIS), and mitochondrial peroxiredoxin-5 (PRDX5). Proteins involved in cellular repair and clearance that differed between the study groups were proteasome activator complex subunit 2 (PSME2), ubiquitin thioesterase (OTUB1), heat shock protein 90 kDa (HSP90AA1), platelet-activating factor acetyl hydrolase IB subunit alpha (PAFAH1B1), heat shock protein 70 kDa (HSPA4), NSFL1 cofactor p47 (NSF1C), and Rho GDP-dissociation inhibitor (ARHGDIB). TF proteins involved in the regulation of cytoskeleton stability that differed between the study groups were myosin light polypeptide 6 (MYL6), destrin (DSTN), and tropomyosin alpha-4 chain (TPM4). TF proteins involved in inflammation and cytoprotection that differed in expression between the two groups included SPARC-like protein 1 (SPARCL1), immunoglobulin J chain (JCHAIN), chitinase-3-like protein (CHI3L2), mucin-5AC (MUC5AC), immunoglobulin heavy variable 5–51 (IGHV5–51), triosephosphate isomerase (TPI1), glucose-6-phosphate isomerase (GPI), alpha-1-antitrypsin (SERPINA1), fibrinogen alpha chain (FGA), prolactin-inducible protein (PIP), mammaglobin (SCGB2A1), immunoglobulin kappa light chain (IGK), lipocalin-1 (LCN1), lysozyme C (LYZ), and deleted in malignant brain tumors 1 protein (DMBT1).

## Discussion

New biomarkers and tests are needed to help identify individuals in the early stages of neurodegenerative diseases, as disease-specific markers are currently lacking.^6,10^ TF proteins are a potential source of biomarkers for many neurodegenerative diseases,^10^ but little is known about the changes in TF protein expression that accompany MCI. In this study, we found 33 TF proteins with a broad range of cellular functions that had significantly altered expression in persons with MCI compared with cognitively healthy controls. We roughly categorized them as proteins involved in or regulating oxidative stress, inflammation, cytoskeleton stability, and protein repair and clearance.

### Proteins related to oxidative stress

Altered expression levels of oxidative stress-related proteins PRDX5, NP1L4, BBOX1, TPIS, PYGL, CST1, CST3, RNASET2, and RNASE4 were observed in TF collected from the MCI group relative to controls. Oxidative stress develops when the protection mechanism (antioxidants) against reactive oxygen species (ROS) production is disturbed.^21,22^

PRDX5 is an important antioxidant protein expressed in mitochondria. PRDX5 is reported to reduce LPS-mediated microglia activation, protect the neurons against ROS, and reduce glutamate-induced cell death.^22^ In our study, mitochondrial PRDX5 was downregulated in the MCI group, and it was the only protein with antioxidant properties. Decreased expression of PRDX5 may contribute to the development of oxidative stress and increase cellular damage in the MCI stage.

The most prominent response was seen in NP1L4 protein, which was upregulated >8-fold in the MCI group. NP1Ls, including NP1L4, regulate the nucleocytoplasmic shuttle via diacylglycerol kinases.^23^ NP1L proteins are thought to be involved in cellular protection against cytotoxicity. Thus, increased levels of NP1Ls may indicate increased cellular cytotoxicity.^24^ The functions of NP1L4 are poorly known in the literature. To the best of our knowledge, the function of NP1L4 has not been linked to MCI or AD previously except in our recently published study.^25^ We think that NP1L4 may be a putative candidate biomarker in MCI.

BBOX1 is a cytosolic enzyme involved in the carnitine synthesis cascade. Carnitine is an essential metabolite for mitochondria and involved in the process of energy metabolism^26^ and the transfer of peroxisomal oxidation products to the mitochondria.^27^ Demand for carnitine is speculated to increase during metabolic stress, and its activity is associated with aging processes.^28^ BBOX1 was highly upregulated in the MCI group and may be linked to increased metabolic stress in the MCI stage.

TPIS regulates the glycogen pathway by participating in glycolytic flow and energy production.^29^ The function of TPIS is highly vulnerable for simultaneous effects of increased oxidative stress and nitric oxide in cellular milieu and causes irreversible changes in TPIS protein structure and loss of function.^30^ Functional deficiency of TPIS enzyme interrupts glycolysis and evokes brain dysfunction and memory loss during neurodegeneration.^29^ TPIS was upregulated in the MCI group, indicating increased production of the protein. Overproduction of TPIS enzyme may also be a compensatory effect against a harmful cell milieu. A limitation of this study is that we do not know the TPIS enzyme activity in the analyzed material.

PYGL is glycogen phosphorylase expressed in the liver. Glycogen phosphorylases are the enzymes that regulate glycogen mobilization in their target tissues: liver, muscle, or brain. PYGL is important for maintaining a constant blood glucose level.^31^ PYGL was upregulated in the MCI group, which may be a response to altered energy metabolism.

We also detected downregulation of CST1 and upregulation of CST3 in the MCI group. Both proteins inhibit cathepsin proteins (cellular proteases), regulate the inflammatory process, and play a protective role in the reduction of oxidative stress.^32–34^ CST1 is known to inhibit the function of cathepsin A and B proteins, which are abundant lysosomal proteases involved in the protein quality system by modulating the rate of protein aggregation. Decreased CST1 levels result in increased amounts of cathepsins and lead to increased protease activity.^32^ CST3 protein is known to inhibit the function of cathepsin D. CST3 is expressed in all cells and secreted in many body fluids. According to previous studies, an increased amount of cathepsin D is linked to AD,^33^ and the amount of CST3 has been reported to increase in response to inflammation and oxidative stress. In the brain in AD, CST3 colocalizes with Aβ peptide^34^ and inhibits the enzymes involved in the degradation of Aβ peptide into toxic Aβ aggregates.^35^ CST3 has already been recognized as a potential biomarker in AD.^32^ Altered protein function of both cathepsins may contribute to oxidative stress, protein aggregation, and the development of inflammation.

Two RNA-binding proteins were upregulated in the MCI group, RNASET2 and RNASE4. Both proteins regulate protein synthesis by controlling RNA longevity.^36,37^ Dysregulation of RNA-binding proteins is widely thought to have a detrimental impact on protein synthesis.^36,37^ However, the exact role of these two proteins during MCI, AD, or other forms of dementia is not known. Increased RNASET2 is speculated to indicate increased ROS production and apoptosis.^38^

### Protein quality control system (PQC) and cytoskeleton stability

We found seven proteins involved in the protein quality control system (PQC) that were altered in MCI: PSME2, OTUB1, HSP90AA1, HSPA4, PAFAH1B1, ARHGDIB, and NSF1C. The PQC includes several cellular pathways important for maintaining protein homeostasis and the degradation and removal of abnormal proteins. An increased amount of misfolded and aggregating proteins is thought to indicate dysfunction in the PQC system.^39,40^

Heat shock proteins HSP90AA1 and HSPA4 are chaperone proteins. Chaperones mediate refolding and degradation of misfolded proteins and prevent protein aggregation.^41^ In AD, HSP90AA1 and HSPA4 form a multichaperone complex with phosphorylated tau.^42^ Given the increased markers of oxidative stress in TF alongside the downregulation of both chaperones, we can interpret this data as indicative of a disrupted protein repair system in MCI.

Ubiquitination is a common post-translational modification that occurs during protein degradation. Tagging misfolded and aggregated proteins with the ubiquitin molecule determines the fate of a protein and starts its transportation to the degradation pathway.^42^ The ubiquitin-proteasome system and the transportation of misfolded proteins includes several enzymes.^43^ PAFAH1B1 links ubiquitin to a substrate, whereas OTUB1 removes the ubiquitin tag from the substrate protein. OTUB1 has also been reported to control apoptosis and the accumulation of tau proteins.^44,45^ PAFAH1B1 has also been shown to support synaptic function and the plasticity of hippocampal CA1 neurons.^46^ PAFAH1B1 was downregulated and OTUB1 upregulated, which may indicate alterations in ubiquitin tagging and an increased number of misfolded proteins in the MCI stage.

PSME2 is a proteasome activator subunit with protective properties against protein aggregation and oxidative stress.^47^ NSFL1 is a cofactor protein involved in the transportation of misfolded protein for proteasomal degradation.^48^ ARHGDIB protein is a Rho GDP-dissociation inhibitor 2 and reported to be involved in Aβ metabolism and NFT production by regulating RhoGTP enzyme activity in AD and vascular dementia.^49^ In the present study, upregulated PSME2 and downregulated NSFL1 and ARHGDIB may indicate an increased need for protection against aggregated proteins already in the MCI stage.

Furthermore, three of the altered proteins (DSTN, TPM4, and MYL6) regulate the function of actin filaments. Actin is important for cell growth, differentiation, division, membrane organization, and motility.^50^ DSTN is an actin-depolymerizing factor, and its increased expression is linked to increased levels of cytoplasmic rod-shaped bundles of filaments composed of ADF/cofilin-actin complex. In AD, increased rods are associated with synaptic dysfunction, disrupted actin dynamics, blocked transport, and loss of mitochondrial membrane potential.^51^ TMP4 is a postsynaptic protein expressed in the growth cones of neurons and formation areas of neurites that regulates actin filament functions. Actin filaments and actin binding proteins are important for the development of fully functional synapses.^52^ MYL6 is a component of myosin, which is also associated with disulfidptosis, a cell death mechanism. Upregulation of this protein may indicate disruptions in the neuronal cytoskeleton, as well as increased cell death in AD.^53^ We found that TMP4 was downregulated and both DSTN and MYL6 upregulated in the MCI group. These results may indicate that similar kinds of disruptions in the function of the cytoskeleton and formation of synapses as reported in AD occur already in the MCI stage.

### Inflammation

TF consists of a wide variety of proteins responsible for the homeostasis of the ocular surface, its nutritional status, antimicrobial function of the eyes, and immunological responses,^54^ including PIP, LCN1, LYZ, DMBT1, SCGB2A1, and MUC5AC.^55,56^ PIP, LCN1, LYZ, DMBT1, and SCGB2A1, which are all produced by the lacrimal glands, were downregulated. Earlier studies reported that PIP, LYZ, and LCN1 are downregulated in AD, and it was speculated that the functions of the lacrimal glands may be disturbed in AD.^7,8^

PIP was the most robustly downregulated protein in the MCI group. PIP plays many roles in the regulation of proteins and enzyme activities and is involved in microglia-mediated neuroinflammation.^57,58^ PIP levels are dependent on the production of multifunctional hormone prolactin (PRL). PRL also plays important roles in the central nervous system (CNS) and is produced by the lacrimal glands.^58^ PRL regulates several brain functions, neurodegeneration, and inflammatory and anti-inflammatory pathways.^58^ PRL levels have been shown to correlate with hippocampal synaptic loss and cognitive dysfunction in patients with type 2 diabetes. Type 2 diabetes is an independent risk factor for AD.^59,60^ Furthermore, decreased PRL levels have been reported in many neurodegenerative diseases,^57^ and the serum PLR concentration has been reported to correlate with TF quality.^58^ Our results support the hypothesis that the function of the lacrimal glands may be disturbed in AD. It seems that lacrimal gland function and its products may be affected already in the MCI stage.

Another eight TF proteins found in the MCI group may be associated with inflammation: SPARCL1, GPI/NLK, FGA, CHI3L2, JCHAIN, IGHV5-51, and IGK. SPARCL1 is an extracellular matrix protein produced by astrocytes. In the brain, it has been shown to play an important role in synapse formation, maturation, pruning, and plasticity.^61,62^ SPARCL1 has been reported to play a protective role in tissue repair and synaptic reorganization.^61^ Altered SPARCL1 expression is also associated with AD and aging. SPARCL1 has been suggested to accelerate AD pathogenesis and be a link to neuroinflammation and altered brain structure and function.^63^ SPARCL1 was upregulated in the TF from the participants in the MCI group. Our findings may indicate disruptions in synapse formation or the presence of inflammation already in the MCI stage.

FGA is one of the fibrinogen coagulation factors capable of converting fibrins into clots and starting the coagulation cascade in response to injury.^64^ Fibrinogen is also an important regulator in the brain. In AD, the presence of Aβ peptide in the brain is thought to induce the formation of abnormal fibril clots that contribute to the development of vascular abnormalities.^65^ Increased levels of fibrinogens are also associated with inflammation via activation of microglia and inhibition of neurite outgrowth.^66,67^ In the present study, FGA was upregulated in the MCI group. Fibrinogens have been considered as candidate biomarkers of vascular abnormalities and inflammation. Our findings support a hypothesis that vascular abnormalities are already developed in the MCI stage.

GPI/NLK is a multifunctional protein, the name of which is dependent on the location where the protein is expressed. Due to the neurotropic function of NLK and the link to AD, we assume that the protein in our experiment may be NLK. In AD, NLK is reported to be expressed in the brain near Aβ plaques and in Aβ-laden vessels. NLK has also been reported to have neuroprotective functions against cell death induced by Aβ peptide in AD.^68^ In earlier studies, overproduction of NLK in cerebrospinal fluid was considered a potential biomarker for recognizing vascular amyloid depositions in patients with amnestic MCI and AD.^68^ We anticipate that upregulated NLK may indicate the presence of Aβ plaques and an increased need for neuroprotection in MCI.

The chitinase-like protein family includes two members, CHI3L1 (YLK-40) and CHI3L2, both of which have been reported to be upregulated in amyotrophic lateral sclerosis and AD.^68–70^ The function of CHI3L2 is not as well described in the literature as the function of CHI3L1, which is a well-known biomarker candidate. However, CHI3L2 is considered a potential fluid biomarker of neuroinflammation.^70^ We detected upregulated CHI3L2 protein in the MCI group, indicating the presence of inflammation.

SERPINA1 is a soluble glycoprotein produced by hepatocytes that has been reported to have anti-inflammatory, anti-infective, and tissue-protective properties. In the CNS, SERPINA1 is associated with inflammation and microglia activation during AD.^71,72^ SERPINA1 is classified as a potential biomarker of memory decline. In patients with type 2 diabetes, the levels of SERPINA1 strongly correlate with high-risk genes for AD. In addition, the levels of plasma SERPINA1 have been reported to be significantly increased in patients with type 2 diabetes who also had MCI.^5^ In the present study, SERPINA1 was upregulated in MCI and may indicate the presence of inflammation.

Downregulated IGK and upregulated JCHAIN and IGHV5-5 proteins in the MCI group may be linked to ongoing immune activation and reduced protective properties. Secretory IgA is the main immunoglobulin in TF,^73^ and the level of IgA is dependent on the amount of subunit protein IGK and JCHAIN protein, which regulate the formation of immunoglobulin IgA and IgM.^74^ IGHV5-51 has been associated with intestinal lesions, immune cell infiltration, and celiac disease.^75^

A strength of this study is that the participants were carefully characterized both neurologically and ophthalmologically. A limitation of the study is the relatively small sample size. To overcome this, we can link the present data to our recently published proteomics data from cognitively healthy controls and patients with AD.^25,76,77^ From 33 significantly altered TF proteins in the MCI group, 18 were significantly altered in our previous study in AD group and 15 had a non-significant response in the AD group but changed in the same direction as in the MCI group, with the exception of GDIR2 and IGK.^25,76,77^ Therefore, TF proteins appear to be similarly altered in both MCI and AD. An interesting question arises as to why some proteins exhibit a milder response in AD than in MCI. We speculate that this could be attributed to the effects of AD medication. Further studies are warranted to shed light on the role of these medications.

## Conclusion

In this study, we demonstrated that the expression of 33 TF proteins in TF was altered in persons with MCI in a rather similar manner as previously reported in patients with AD. Our study provides new information about TF protein expression in MCI, risk stage for dementia, and raises new research questions. The findings encourage us to continue our search for easy-to-collect and non-invasive TF biomarkers to detect the early stages of dementia. TF proteins hold promise as a potential source of new biomarker molecules.
